# Predictive factors of immune tolerance treatment response in severe haemophilia A patients with inhibitors: A real‐world report from a single centre, mixed retrospective‐prospective long‐term study

**DOI:** 10.1111/hae.13660

**Published:** 2019-01-03

**Authors:** Saturnino Haya, Carlos Solano, Ana Rosa Cid, Bienvenida Argilés, David Hervás, Felipe Querol, Santiago Bonanad, Pilar Casaña

**Affiliations:** ^1^ Hemostasis and Thrombosis Unit, Hematology Service Hospital Universitari i Politècnic La Fe Valencia Spain; ^2^ Hematology Service Hospital Clínico de Valencia Valencia Spain; ^3^ Department of Medicine Universidad de Valencia Valencia Spain; ^4^ Pediatric Hematology Unit Hospital Universitari i Politècnic La Fe Valencia Spain; ^5^ Unit of Data Science, Biostatistics and Bioinformatics Instituto de Investigación Sanitaria La Fe Valencia Spain; ^6^ Departament of Physiotherapy Universidad de Valencia Valencia Spain

Dear Editor,

The appropriate management of patients with inhibitors represents the main challenge for physicians who specialize in haemophilia. Immune tolerance induction (ITI) is the primary therapeutic strategy for achieving inhibitor eradication.^1^ ITI represents an intensive and continuous exposure to FVIII until a patient gains complete or partial tolerance against the factor. To contribute to the data from an experienced centre in managing haemophilic patients, the present mixed retrospective and prospective study was aimed to analyse the association between the ITI success rate with a series of clinical variables. The medical records of severe haemophilia A patients from a Congenital Coagulopathies Unit, who started an ITI regimen between March 1980 and July 2015, were reviewed. The study was conducted in accordance with the Declaration of Helsinki and Good Clinical Practice.

Eligible subjects included both children and adults diagnosed with severe haemophilia A (FVIII:C <1%) and treated for primary or rescue ITI with plasma‐derived FVIII concentrates (pdFVIII), either purified FVIII or von Willebrand factor (VWF)‐containing (pdFVIII/VWF), or with recombinant (rFVIII) concentrates. Rescue ITI was defined as the ITI treatments undergone after failure of the primary ITI course. The definitions of ITI success and failure were generally consistent with those currently in use.^1^ The time to outcome was measured from initiation of ITI until achievement of success (complete or partial), failure or rescue ITI. The decision of whether ITI was a failure, or to continue ITI treatment longer, was made according to the physician's discretion.

For pharmacokinetic measurements, after 3 days of infused FVIII washing, FVIII:C levels were determined (“pre”). Then, 50 IU/kg FVIII was administered and FVIII:C was determined again after 15 minutes (“post”), 1, 2, 6, 24 and 48 hours (although last 7 years, measurements were taken at three time points: pre, post and 48 hours).

For association analyses, a L1‐penalized logistic regression model, LASSO (Least Absolute Shrinkage and Selection Operator), was used. Those variables not penalized to zero were considered as being associated with ITI success. Peak titre was log‐transformed before the analyses because of its high right skewness. The use of penalized models is required in cases such as in this study, in which the number of variables was high in relation to the number of assessments.

Kaplan‐Meier survival analysis was constructed for the time elapsed in the percentage of patients: (a) reaching inhibitor elimination; (b) reaching a normal FVIII recovery and (c) reaching a normal half‐life of infused FVIII. Discrimination by infused FVIII dose (<100 IU/kg/d; ≥100 IU/kg/d) was also made. The generalized Wilcoxon test was used for comparison. Software R (The R Foundation, Vienna, Austria) version 3.2.1 was used for calculations and analysis.

Results showed that 26 patients started an ITI course during the study period. Of these, three patients are still under treatment while 23 ended primary ITI and were therefore evaluated. Data collected were retrospective in patients who started ITI up to year 2000 (n = 11) and prospective after that year (n = 12 patients). Details of patient characteristics and ITI data are shown in Table 1.

**Table 1:** Immune tolerance induction patient data and outcome of ITI

Patient #	Pre‐ITI data					Primary ITI data									Rescue ITI data	
	Historic titre (BU/mL)	Titre at ITI start (BU/mL)	Age at ITI start (y)	Diagnosis to ITI (mo)	Type of FVIII at inhibitor detection	Peak titre (BU/mL)	Duration (mo)	Haemorrhagic events (n)	Type of FVIII	Dosage (IU/kg)	CVC infection	Bypass agent	Immunomodulation	ITI outcome	Type of FVIII	ITI outcome
1	22	8	25^a^	50^a^	PD	23	3	0	PD	50/d	Yes	No	No	CS		
2	41	6	21^a^	132^a^	PD	41	11	3	PD	50/d	Yes	No	CORT	CS		
3	295^a^	50^a^	10^a^	25^a^	PD	294^a^	28	22	PD	50/d	No	No	CORT & PLPH	CS		
4	86	2	8^a^	46^a^	PD	211^a^	11	13	PD	10 3/wk	Yes	No	No	F		
5	70	32^a^	2	12	PD	32	33	20	PD	50/d	Yes	No	CORT	CS		
6	1075^a^	183^a^	4	32^a^	PD	1075^a^	28	12	PD	50/d	No	No	CORT	F		
7	14	2	8^a^	55^a^	PD	2	13	3	PD	100/d	No	No	CORT	CS		
8	114	1	2	15	PD	53^a^	22	3	PD	100/d	No	No	IG	CS		
9	13	6	4	11	PD	3	35	12	PD	70 /d	Yes	No	CORT	CS		
10	52	52^a^	0	0	REC	276^a^	10	1	REC	200/d	Yes	No	IG	F	PD	PS
11	5	5	2	1	REC	4	31	13	REC	50 3/wk	No	No	No	CS		
12	7	3	3	12	PD	723^a^	10	17	PD	50 3/wk	No	No	No	F	PD	CS
13	2	2	2	5	REC	1	4	0	REC	50 3/wk	No	No	No	CS		
14	21	2	1	14	PD	150	12	0	PD	200/d	No	No	No	CS		
15	4	2	4	0	PD	2	2	2	REC	50 3/wk	Yes	No	No	F	PD	CS
16	13	4	2	3	REC	3	9	2	REC	50 3/wk	No	Yes	No	CS		
17	11	4	1	5	REC	209^a^	13	16	REC	50 3/wk	No	Yes	No	F	PD	PS
18	134	5	3	10	REC	340^a^	24	13	REC	50 3/wk	No	Yes	No	F	PD	Ongoing
19	29	1	2	9	REC	19	10	1	PD	133/d	No	No	No	CS		
20	7	4	0	0	REC	3	5	1	REC	200/d	No	Yes	No	F		
21	11	11^a^	1	1	REC	9	11	9	PD	80 3/wk	No	Yes	No	CS		
22	10	10	1	1	REC	3	12	2	REC	200 3/wk	No	No	No	F	PD	CS
23	24	7	1	0	REC	24	5	4	PD	100 3/wk	No	Yes	No	F	PD	Ongoing
Median	21.0	4.6	2.0	10.0		23.7	12.0	3.0								
Q1	10.2	2.0	1.0	1.0		2.8	9.4	1.5								
Q3	61.0	8.8	4.0	20.0		210.0	22.9	13.0								

CORT, corticoids; CS, complete success; CVC, central venous catheter; d, days; F, failure; IG, immune globulin; ITI, immune tolerance induction; mo, months; PD, plasma‐derived; PLPH, plasmapheresis; PS, partial success; Q1, first quartile; Q3, third quartile; REC, recombinant; wk, weeks; y, years.

a Denotes a risk factor for poor response to ITI: (a) age at start of ITI >7 y old; (b) historical inhibitor peak titre >200 BU; (c) time between inhibitor diagnosis and start of ITI >2 y; and (d) inhibitor titre at start of ITI >10 BU.

Just over half of the patients (n = 13; 57%) started ITI within 1 year after inhibitor diagnostic (47% of complete success [CS]), while in nine patients (39%) the lapse took between 1 and 5 years, and one patient showed an extreme value of 11 years. Eighteen patients (78%) were <5 years old at the time of ITI initiation (50% of CS). The majority of the patients (n = 18; 78%) showed an inhibitor titre <10 BU/mL at start of ITI (55% of CS) but five of them had titre ≥10 BU/mL (60% of CS). Overall, primary ITI success was 57% (13/23 patients), which was lower than that shown in previous studies ranging 63%‐100%.^2^, ^3^, ^4^, ^5^, ^6^, ^7^ A possible reason may be the inclusion of all screened patients in the study, thus mimicking a group with intention of treatment, which has shown a lower success rate. Similarly, premature changes of product type or dose regimen in some patients as well as the high historical inhibitor peak titre in our population could have a role in reducing the chances of success. Nevertheless, less strict criteria than ours for reporting an outcome of successful ITI have been described in other studies^6^, ^8^ and in a registry.^9^

The median time of ITI treatment was 11.4 months (Q1, Q3: 9, 24). Fourteen patients (61%) received pdFVIII (13 pdFVIII/VWF and 1 purified FVIII) for ITI. There was a higher rate of CS in patients treated with pdFVIII (10/14; 71%) rather than those treated with rFVIII (3/9; 33%). Interestingly, the percentage of patients with at least one risk factor for poor response to ITI^10^ was higher in those pdFVIII‐treated than in rFVIII‐treated (64%, 9/14 vs 33%, 3/9, respectively), as seen in Table 1.

FVIII dosage was <100 IU/kg/d in 17 patients, 10 of them treated with pdFVIII (71%) and seven treated with rFVIII (78%). CS was reached in 8/17 (47%) patients treated with a dose <100 IU/kg/d, and in 5/6 (83%) patients treated with a dose ≥100 IU/kg/d.

The FVIII infusion with a central venous catheter (CVC) was performed in 30% (n = 7) of the patients and almost all of them (6/7; 86%) suffered infection episodes. Haemorrhagic episodes during ITI were common (n = 20; 80%) and recurrent (median: 3; Q1, Q3: 1.5, 13) among all patients. Of those, 26% (n = 6) used a bypassing agent as prophylaxis. Those patients with a FVIII daily dose ≥100 IU/kg showed the lowest number of haemorrhagic episodes (median: 1.5; Q1, Q3: 1, 3). Eight patients (35%) received concomitant medication, and all of them achieved CS. Immunomodulatory agents were the most frequently prescribed (Table 1).

Rescue ITI was performed with pdFVIII/VWF in almost all failure patients (8/10) after an initial failure, although data from one patient were not available for analysis due to withdrawal after ITI initiation (Table 1). All rescue patients were children (<9 years old) who required between two and five rescue ITI courses to achieve CS (3/7; 43%) or partial success (PS) (2/7; 29%). Results of the two remaining patients were not considered in the success calculation, as their treatment was unfinished by the end of the study. Remarkably, the overall success rate, after primary or rescue ITI, was 86% (n = 18/21) despite several subjects had factors for poor response to ITI as reported in registries.^5^, ^9^

Statistical analysis by LASSO regression reported three variables to be associated with the probability of ITI success. Two variables showed a negative association: infusion of rFVIII (odds ratio [OR] = 0.51) and inhibitor peak titre during ITI (OR = 0.88; Figure 1). The third variable associated with outcome was pdFVIII/VWF infusion although the effect was clinically negligible (7 × 10^−14^). However, the estimation for this variable was affected by the fact that only one patient out of the 14 patients receiving pdFVIII concentrate did not receive pdFVIII/VWF.

**Figure 1:**
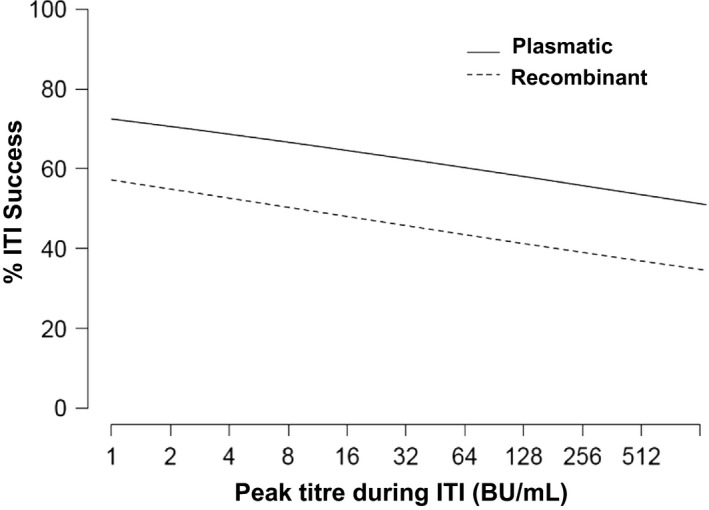
Chart results of the logistic regression with penalization L_1_ (LASSO). Percentage of immune tolerance induction (ITI) success using a plasmatic or a recombinant FVIII concentrate, according to the inhibitor peak titre reached during ITI

The inverse relation of ITI success rate and the inhibitor peak titre during ITI is supported by the results of other reports.^2^, ^9^, ^11^ However, our study did not find a relationship between ITI success and the historical peak titre, nor the titre at ITI start, as previously reported.^3^, ^6^, ^9^, ^12^

The effect of the type of concentrate infused was also highlighted by LASSO. A better outcome resulted in patients treated with pdFVIII rather than rFVIII, consistent with previous publications.^13^, ^14^ However, there is some controversy because the use of rFVIII with a good success rate has also been described.^8^, ^15^ Nevertheless, in all cases, comparisons should be made with caution due to the differences in methodologies, protocols and outcome criteria.

The use of pdFVIII/VWF has been reported to improve ITI success.^7^, ^11^ Although in our study the LASSO analysis yielded an extremely small effect associated with pdFVIII/VWF, such statistical effect could not be quantified with accuracy because only one patient out of 14 was not infused with pdFVIII/VWF.

The FVIII dosages given in this study did not influence the ITI outcome. Personalized doses were used, depending on the peak titre of each patient, similar to the procedures applied by other groups.^12^ The Kaplan‐Meier analysis showed no statistically significant differences in time to success when comparing the two dose groups established (<100 IU/kg/d; ≥100 IU/kg/d).

Other parameters described to may influence ITI outcome but not found in our patient series were as follows: type of mutation, age at ITI start, delay from inhibitor detection of ITI start and infection of the CVC.^5^, ^9^

Although the cohort of patients recruited was relatively small and almost half of the patient data were retrospective, which could be considered a study limitation, it is a real‐world study; the fact that patients were unselected and all data came from a single centre with consistent routine procedures for more than 20 years confers robustness and homogeneity to the data.

In conclusion, in this study, a high ITI success rate of 86% was found in severe haemophilia A patients from a single centre who were screened over a 25‐year period. Moreover, low inhibitor titre peak during ITI and the infusion of pdFVIII were found to be predictors of a higher ITI success rate.

## DISCLOSURES

SH has given lectures at educational symposiums organized by Novo Nordisk, Pfizer, Grifols and Baxter; Advisory boards from Shire. CS has given lectures at educational symposiums organized by Pfizer, Roche, Merck and Astellas. ARC has given lectures at educational symposiums organized by Novo Nordisk, Pfizer, Grifols and Baxter. FQ has received grants from Baxter, Bayer, Pfizer and Novonordis; has given lectures at educational symposiums organized by Novo Nordisk, Pfizer, Baxter and Shire. SB has given lectures at educational symposiums organized by Novo Nordisk, Pfizer, Grifols, Bayer, Roche, LFB, Octapharma, Shire, SOBI and CSL Behring; Advisory boards from Pfizer, Bayer, Roche, Shire, LFB, SOBI and CSL Behring. BA, DH, and PC declare no conflict of interests.

## AUTHORS’ CONTRIBUTION

SH designed the research study, performed the research, recruited the patients and wrote the paper; CS and PC designed the research study and supervised the study; ARC, BA, FQ and SB recruited the patients; and DH analysed the data. All authors read and approved the final manuscript.
